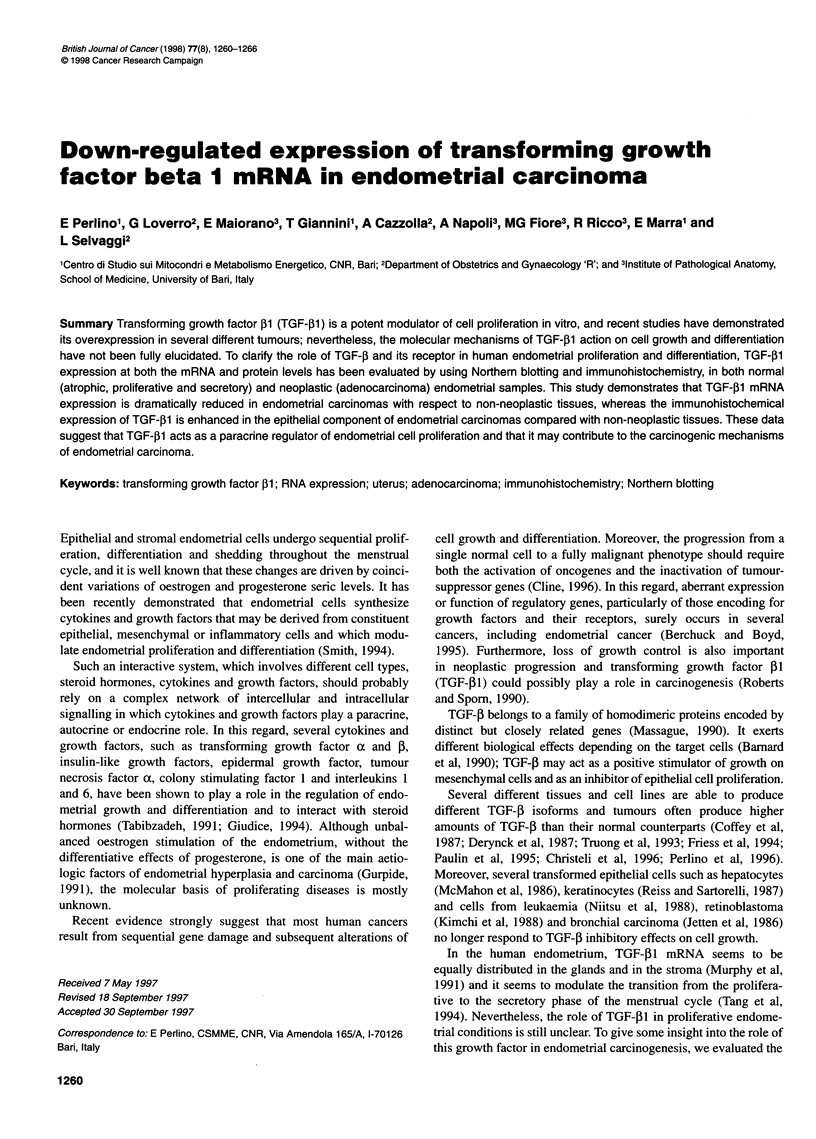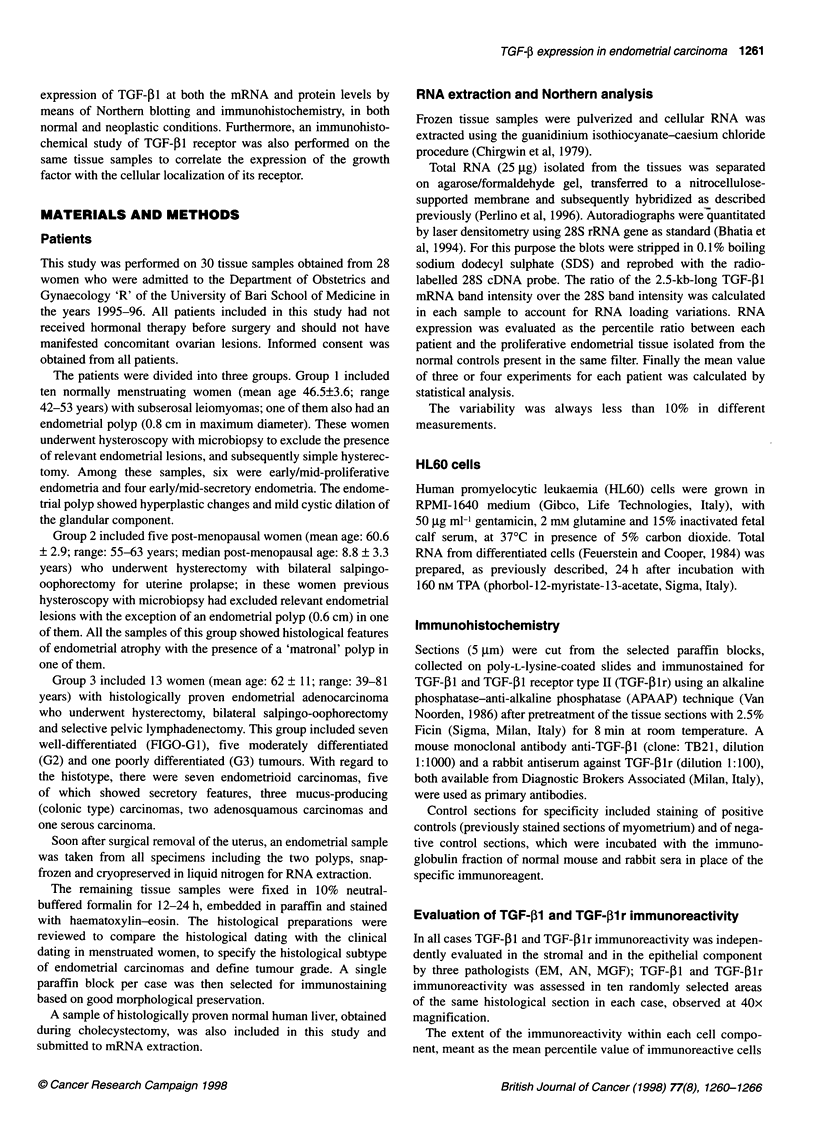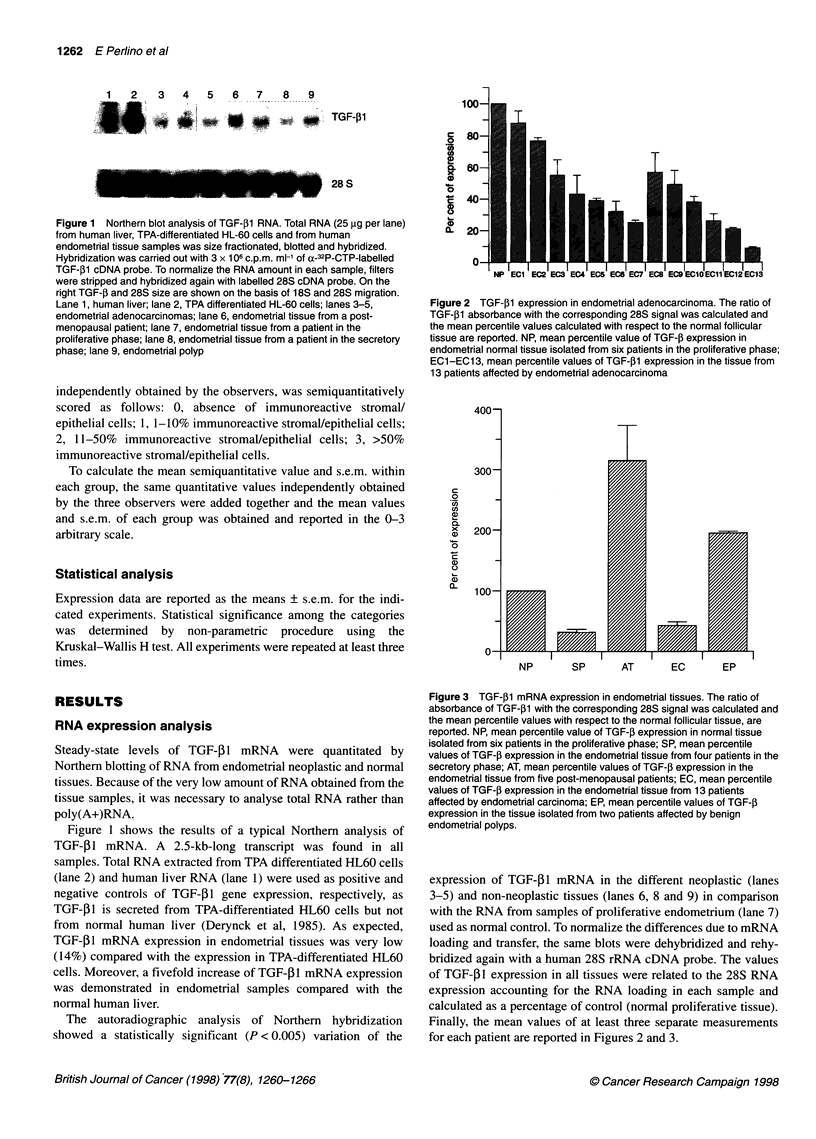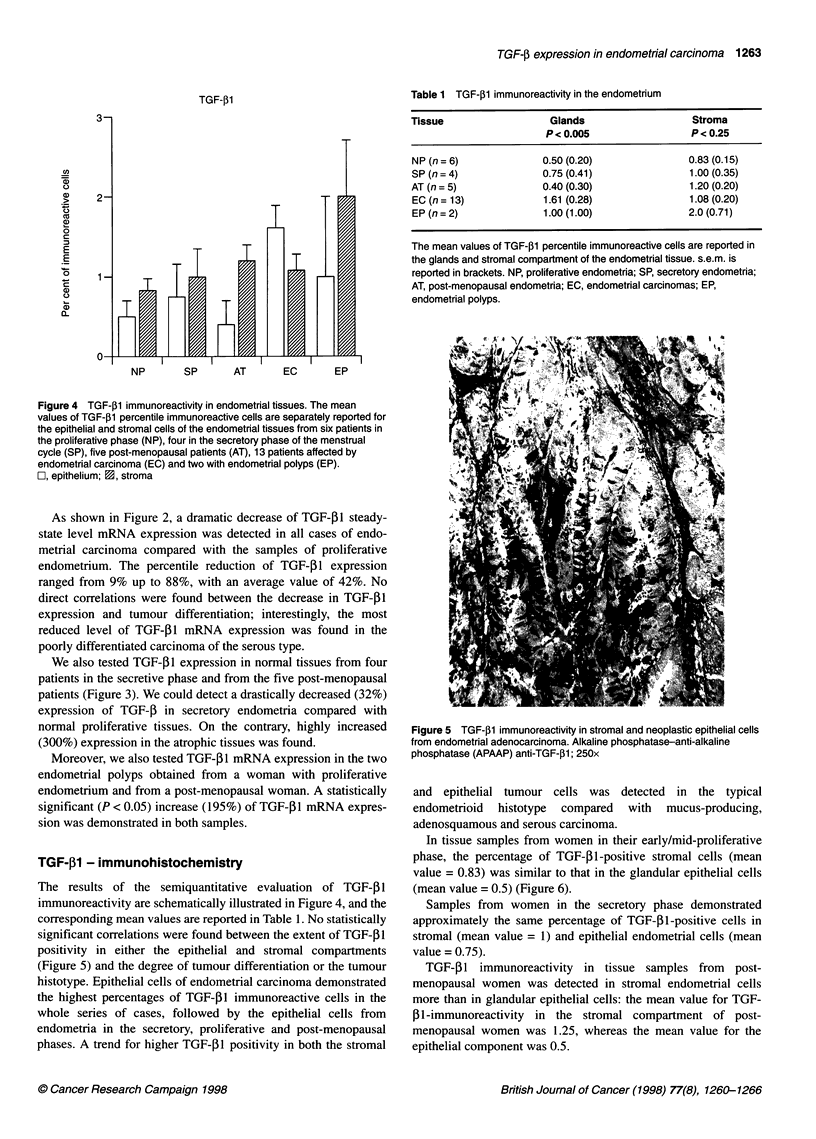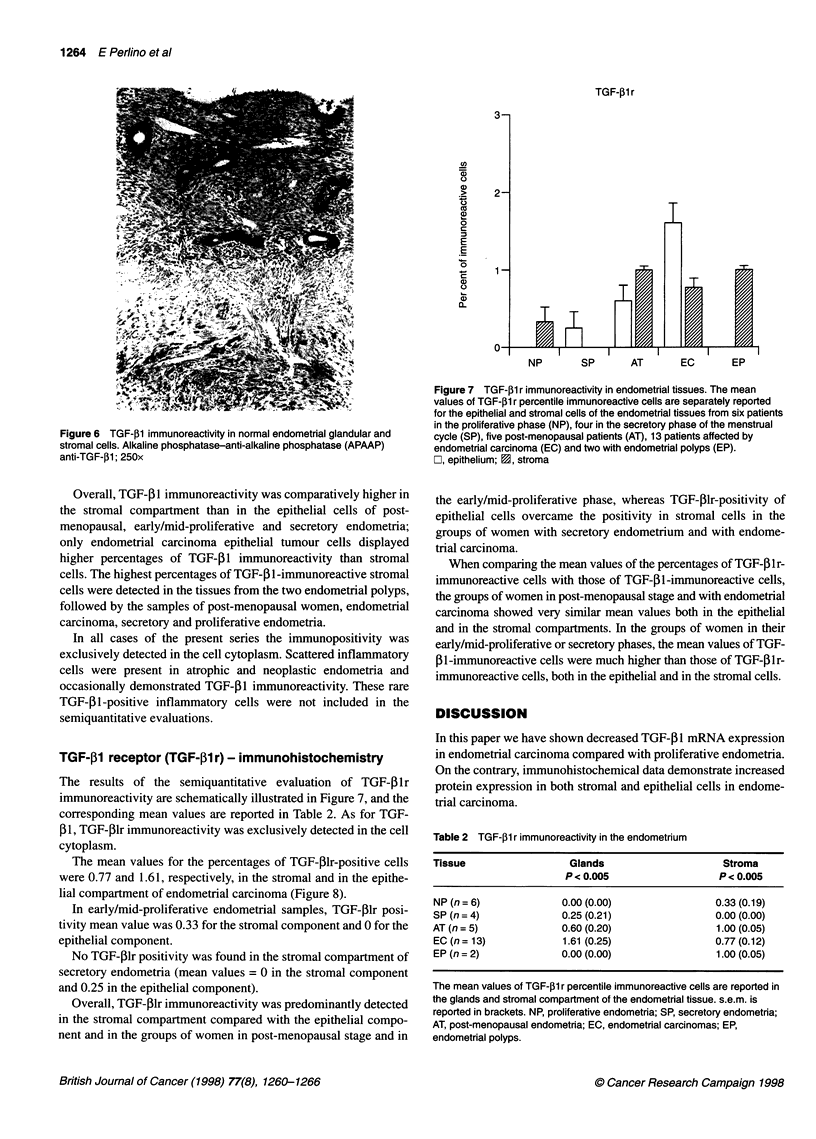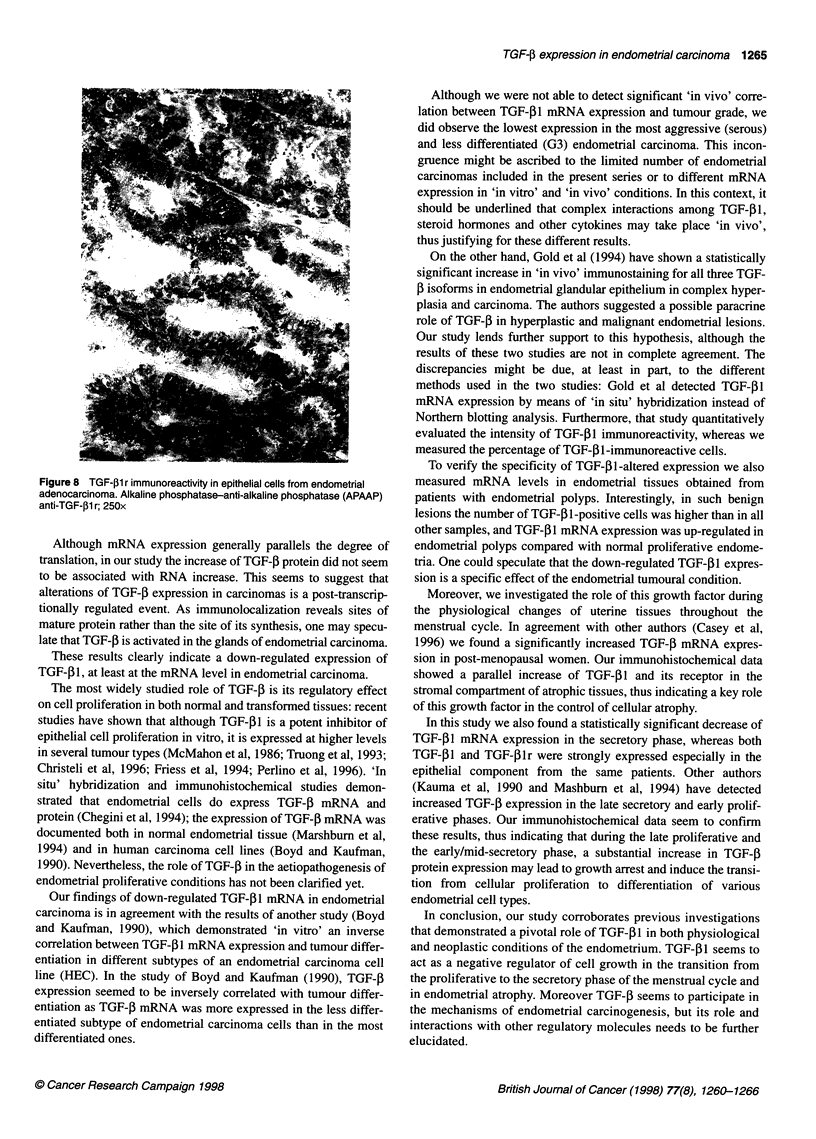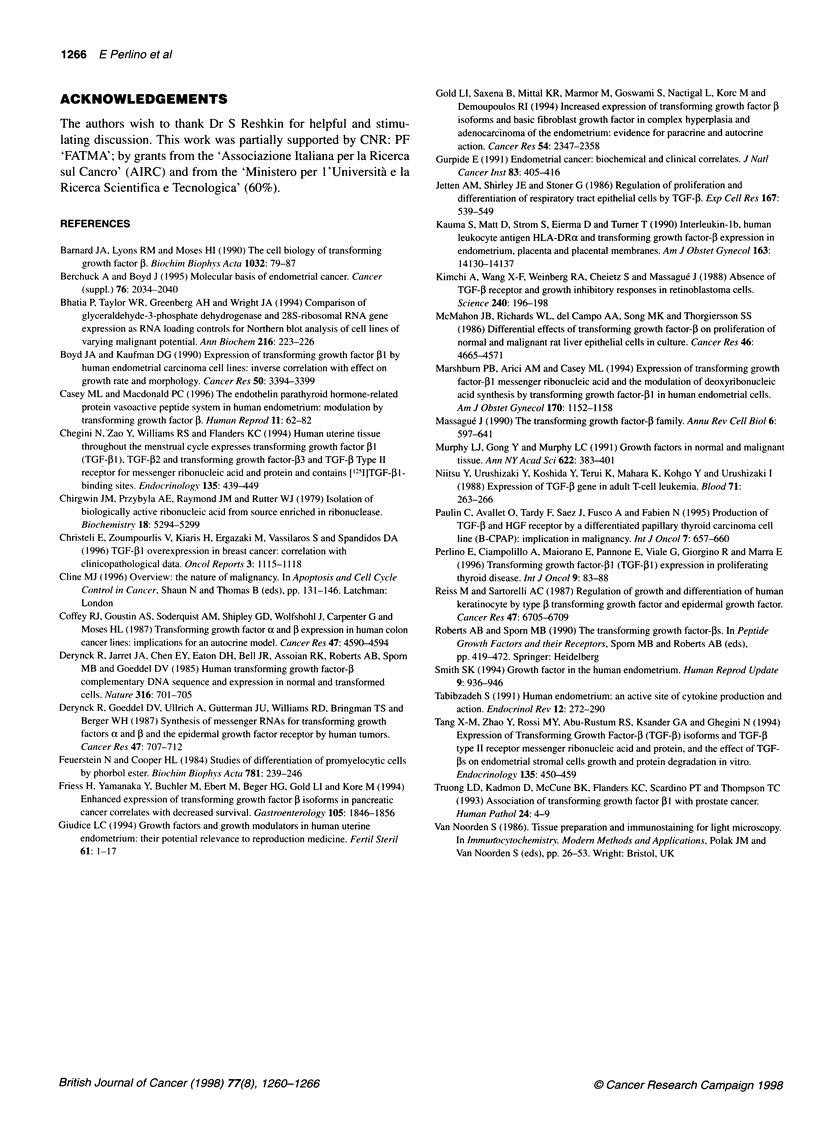# Down-regulated expression of transforming growth factor beta 1 mRNA in endometrial carcinoma.

**DOI:** 10.1038/bjc.1998.211

**Published:** 1998-04

**Authors:** E. Perlino, G. Loverro, E. Maiorano, T. Giannini, A. Cazzolla, A. Napoli, M. G. Fiore, R. Ricco, E. Marra, L. Selvaggi

**Affiliations:** Centro di Studio sui Mitocondri e Metabolismo Energetico, CNR, Bari, Italy.

## Abstract

**Images:**


British Journal of Cancer (1998) 77(8), 1260–1266
© 1998 Cancer Research Campaign

### Down-regulated expression of transforming growth factor beta 1 mRNA in endometrial carcinoma

E Perlino[1], G Loverro[2], E Maiorano[3], T Giannini[1], A Cazzolla[2], A Napoli[3], MG Fiore[3], R Ricco[3], E Marra[1] and L Selvaggi[2]

[1]Centro di Studio sui Mitocondri e Metabolismo Energetico, CNR, Bari; [2]Department of Obstetrics and Gynaecology 'R'; and [3]Institute of Pathological Anatomy, School of Medicine, University of Bari, Italy

**Summary** Transforming growth factor β1 (TGF-β1) is a potent modulator of cell proliferation in vitro, and recent studies have demonstrated its overexpression in several different tumours; nevertheless, the molecular mechanisms of TGF-β1 action on cell growth and differentiation have not been fully elucidated. To clarify the role of TGF-β and its receptor in human endometrial proliferation and differentiation, TGF-β1 expression at both the mRNA and protein levels has been evaluated by using Northern blotting and immunohistochemistry, in both normal (atrophic, proliferative and secretory) and neoplastic (adenocarcinoma) endometrial samples. This study demonstrates that TGF-β1 mRNA expression is dramatically reduced in endometrial carcinomas with respect to non-neoplastic tissues, whereas the immunohistochemical expression of TGF-β1 is enhanced in the epithelial component of endometrial carcinomas compared with non-neoplastic tissues. These data suggest that TGF-β1 acts as a paracrine regulator of endometrial cell proliferation and that it may contribute to the carcinogenic mechanisms of endometrial carcinoma.

**Keywords:** transforming growth factor β1; RNA expression; uterus; adenocarcinoma; immunohistochemistry; Northern blotting

Epithelial and stromal endometrial cells undergo sequential proliferation, differentiation and shedding throughout the menstrual cycle, and it is well known that these changes are driven by coincident variations of oestrogen and progesterone seric levels. It has been recently demonstrated that endometrial cells synthesize cytokines and growth factors that may be derived from constituent epithelial, mesenchymal or inflammatory cells and which modulate endometrial proliferation and differentiation (Smith, 1994).

Such an interactive system, which involves different cell types, steroid hormones, cytokines and growth factors, should probably rely on a complex network of intercellular and intracellular signalling in which cytokines and growth factors play a paracrine, autocrine or endocrine role. In this regard, several cytokines and growth factors, such as transforming growth factor α and β, insulin-like growth factors, epidermal growth factor, tumour necrosis factor α, colony stimulating factor 1 and interleukins 1 and 6, have been shown to play a role in the regulation of endometrial growth and differentiation and to interact with steroid hormones (Tabibzadeh, 1991; Giudice, 1994). Although unbalanced oestrogen stimulation of the endometrium, without the differentiative effects of progesterone, is one of the main aetiologic factors of endometrial hyperplasia and carcinoma (Gurpide, 1991), the molecular basis of proliferating diseases is mostly unknown.

Recent evidence strongly suggest that most human cancers result from sequential gene damage and subsequent alterations of cell growth and differentiation. Moreover, the progression from a single normal cell to a fully malignant phenotype should require both the activation of oncogenes and the inactivation of tumour-suppressor genes (Cline, 1996). In this regard, aberrant expression or function of regulatory genes, particularly of those encoding for growth factors and their receptors, surely occurs in several cancers, including endometrial cancer (Berchuck and Boyd, 1995). Furthermore, loss of growth control is also important in neoplastic progression and transforming growth factor β1 (TGF-β1) could possibly play a role in carcinogenesis (Roberts and Sporn, 1990).

TGF-β belongs to a family of homodimeric proteins encoded by distinct but closely related genes (Massague, 1990). It exerts different biological effects depending on the target cells (Barnard et al, 1990); TGF-β may act as a positive stimulator of growth on mesenchymal cells and as an inhibitor of epithelial cell proliferation.

Several different tissues and cell lines are able to produce different TGF-β isoforms and tumours often produce higher amounts of TGF-β than their normal counterparts (Coffey et al, 1987; Derynck et al, 1987; Truong et al, 1993; Friess et al, 1994; Paulin et al, 1995; Christeli et al, 1996; Perlino et al, 1996). Moreover, several transformed epithelial cells such as hepatocytes (McMahon et al, 1986), keratinocytes (Reiss and Sartorelli, 1987) and cells from leukaemia (Niitsu et al, 1988), retinoblastoma (Kimchi et al, 1988) and bronchial carcinoma (Jetten et al, 1986) no longer respond to TGF-β inhibitory effects on cell growth.

In the human endometrium, TGF-β1 mRNA seems to be equally distributed in the glands and in the stroma (Murphy et al, 1991) and it seems to modulate the transition from the proliferative to the secretory phase of the menstrual cycle (Tang et al, 1994). Nevertheless, the role of TGF-β1 in proliferative endometrial conditions is still unclear. To give some insight into the role of this growth factor in endometrial carcinogenesis, we evaluated the

*Received 7 May 1997*
*Revised 18 September 1997*
*Accepted 30 September 1997*

*Correspondence to:* E Perlino, CSMME, CNR, Via Amendola 165/A, I-70126 Bari, Italy expression of TGF-β1 at both the mRNA and protein levels by means of Northern blotting and immunohistochemistry, in both normal and neoplastic conditions. Furthermore, an immunohistochemical study of TGF-β1 receptor was also performed on the same tissue samples to correlate the expression of the growth factor with the cellular localization of its receptor.

### MATERIALS AND METHODS

### Patients

This study was performed on 30 tissue samples obtained from 28 women who were admitted to the Department of Obstetrics and Gynaecology 'R' of the University of Bari School of Medicine in the years 1995–96. All patients included in this study had not received hormonal therapy before surgery and should not have manifested concomitant ovarian lesions. Informed consent was obtained from all patients.

The patients were divided into three groups. Group 1 included ten normally menstruating women (mean age 46.5±3.6; range 42–53 years) with subserosal leiomyomas; one of them also had an endometrial polyp (0.8 cm in maximum diameter). These women underwent hysteroscopy with microbiopsy to exclude the presence of relevant endometrial lesions, and subsequently simple hysterectomy. Among these samples, six were early/mid-proliferative endometria and four early/mid-secretory endometria. The endometrial polyp showed hyperplastic changes and mild cystic dilation of the glandular component.

Group 2 included five post-menopausal women (mean age: 60.6 ± 2.9; range: 55–63 years; median post-menopausal age: 8.8 ± 3.3 years) who underwent hysterectomy with bilateral salpingo-oophorectomy for uterine prolapse; in these women previous hysteroscopy with microbiopsy had excluded relevant endometrial lesions with the exception of an endometrial polyp (0.6 cm) in one of them. All the samples of this group showed histological features of endometrial atrophy with the presence of a 'matronal' polyp in one of them.

Group 3 included 13 women (mean age: 62 ± 11; range: 39–81 years) with histologically proven endometrial adenocarcinoma who underwent hysterectomy, bilateral salpingo-oophorectomy and selective pelvic lymphadenectomy. This group included seven well-differentiated (FIGO-G1), five moderately differentiated (G2) and one poorly differentiated (G3) tumours. With regard to the histotype, there were seven endometrioid carcinomas, five of which showed secretory features, three mucus-producing (colonic type) carcinomas, two adenosquamous carcinomas and one serous carcinoma.

Soon after surgical removal of the uterus, an endometrial sample was taken from all specimens including the two polyps, snap-frozen and cryopreserved in liquid nitrogen for RNA extraction.

The remaining tissue samples were fixed in 10% neutral-buffered formalin for 12–24 h, embedded in paraffin and stained with haematoxylin–eosin. The histological preparations were reviewed to compare the histological dating with the clinical dating in menstruated women, to specify the histological subtype of endometrial carcinomas and define tumour grade. A single paraffin block per case was then selected for immunostaining based on good morphological preservation.

A sample of histologically proven normal human liver, obtained during cholecystectomy, was also included in this study and submitted to mRNA extraction.

### RNA extraction and Northern analysis

Frozen tissue samples were pulverized and cellular RNA was extracted using the guanidinium isothiocyanate–caesium chloride procedure (Chirgwin et al, 1979).

Total RNA (25 μg) isolated from the tissues was separated on agarose/formaldehyde gel, transferred to a nitrocellulose-supported membrane and subsequently hybridized as described previously (Perlino et al, 1996). Autoradiographs were quantitated by laser densitometry using 28S rRNA gene as standard (Bhatia et al, 1994). For this purpose the blots were stripped in 0.1% boiling sodium dodecyl sulphate (SDS) and reprobed with the radio-labelled 28S cDNA probe. The ratio of the 2.5-kb-long TGF-β1 mRNA band intensity over the 28S band intensity was calculated in each sample to account for RNA loading variations. RNA expression was evaluated as the percentile ratio between each patient and the proliferative endometrial tissue isolated from the normal controls present in the same filter. Finally the mean value of three or four experiments for each patient was calculated by statistical analysis.

The variability was always less than 10% in different measurements.

### HL60 cells

Human promyelocytic leukaemia (HL60) cells were grown in RPMI-1640 medium (Gibco, Life Technologies, Italy), with 50 μg $ml^{-1}$ gentamicin, 2 mM glutamine and 15% inactivated fetal calf serum, at 37°C in presence of 5% carbon dioxide. Total RNA from differentiated cells (Feuerstein and Cooper, 1984) was prepared, as previously described, 24 h after incubation with 160 nM TPA (phorbol-12-myristate-13-acetate, Sigma, Italy).

### Immunohistochemistry

Sections (5 μm) were cut from the selected paraffin blocks, collected on poly-L-lysine-coated slides and immunostained for TGF-β1 and TGF-β1 receptor type II (TGF-β1r) using an alkaline phosphatase–anti-alkaline phosphatase (APAAP) technique (Van Noorden, 1986) after pretreatment of the tissue sections with 2.5% Ficin (Sigma, Milan, Italy) for 8 min at room temperature. A mouse monoclonal antibody anti-TGF-β1 (clone: TB21, dilution 1:1000) and a rabbit antiserum against TGF-β1r (dilution 1:100), both available from Diagnostic Brokers Associated (Milan, Italy), were used as primary antibodies.

Control sections for specificity included staining of positive controls (previously stained sections of myometrium) and of negative control sections, which were incubated with the immunoglobulin fraction of normal mouse and rabbit sera in place of the specific immunoreagent.

### Evaluation of TGF-β1 and TGF-β1r immunoreactivity

In all cases TGF-β1 and TGF-β1r immunoreactivity was independently evaluated in the stromal and in the epithelial component by three pathologists (EM, AN, MGF); TGF-β1 and TGF-β1r immunoreactivity was assessed in ten randomly selected areas of the same histological section in each case, observed at 40× magnification.

The extent of the immunoreactivity within each cell component, meant as the mean percentile value of immunoreactive cells

© Cancer Research Campaign 1998

**Figure 1** Northern blot analysis of TGF-β1 RNA. Total RNA (25 μg per lane) from human liver, TPA-differentiated HL-60 cells and from human endometrial tissue samples was size fractionated, blotted and hybridized. Hybridization was carried out with $3 \times 10^6$ c.p.m. $ml^{-1}$ of α-$^{32}$P-CTP-labelled TGF-β1 cDNA probe. To normalize the RNA amount in each sample, filters were stripped and hybridized again with labelled 28S cDNA probe. On the right TGF-β and 28S size are shown on the basis of 18S and 28S migration. Lane 1, human liver; lane 2, TPA differentiated HL-60 cells; lanes 3–5, endometrial adenocarcinomas; lane 6, endometrial tissue from a post-menopausal patient; lane 7, endometrial tissue from a patient in the proliferative phase; lane 8, endometrial tissue from a patient in the secretory phase; lane 9, endometrial polyp independently obtained by the observers, was semiquantitatively scored as follows: 0, absence of immunoreactive stromal/epithelial cells; 1, 1–10% immunoreactive stromal/epithelial cells; 2, 11–50% immunoreactive stromal/epithelial cells; 3, >50% immunoreactive stromal/epithelial cells.

To calculate the mean semiquantitative value and s.e.m. within each group, the same quantitative values independently obtained by the three observers were added together and the mean values and s.e.m. of each group was obtained and reported in the 0–3 arbitrary scale.

### Statistical analysis

Expression data are reported as the means ± s.e.m. for the indicated experiments. Statistical significance among the categories was determined by non-parametric procedure using the Kruskal–Wallis H test. All experiments were repeated at least three times.

### RESULTS

### RNA expression analysis

Steady-state levels of TGF-β1 mRNA were quantitated by Northern blotting of RNA from endometrial neoplastic and normal tissues. Because of the very low amount of RNA obtained from the tissue samples, it was necessary to analyse total RNA rather than poly(A+)RNA.

Figure 1 shows the results of a typical Northern analysis of TGF-β1 mRNA. A 2.5-kb-long transcript was found in all samples. Total RNA extracted from TPA differentiated HL60 cells (lane 2) and human liver RNA (lane 1) were used as positive and negative controls of TGF-β1 gene expression, respectively, as TGF-β1 is secreted from TPA-differentiated HL60 cells but not from normal human liver (Derynck et al, 1985). As expected, TGF-β1 mRNA expression in endometrial tissues was very low (14%) compared with the expression in TPA-differentiated HL60 cells. Moreover, a fivefold increase of TGF-β1 mRNA expression was demonstrated in endometrial samples compared with the normal human liver.

The autoradiographic analysis of Northern hybridization showed a statistically significant ($P < 0.005$) variation of the

**Figure 2** TGF-β1 expression in endometrial adenocarcinoma. The ratio of TGF-β1 absorbance with the corresponding 28S signal was calculated and the mean percentile values calculated with respect to the normal follicular tissue are reported. NP, mean percentile value of TGF-β expression in endometrial normal tissue isolated from six patients in the proliferative phase; EC1–EC13, mean percentile values of TGF-β1 expression in the tissue from 13 patients affected by endometrial adenocarcinoma

**Figure 3** TGF-β1 mRNA expression in endometrial tissues. The ratio of absorbance of TGF-β1 with the corresponding 28S signal was calculated and the mean percentile values with respect to the normal follicular tissue, are reported. NP, mean percentile value of TGF-β expression in normal tissue isolated from six patients in the proliferative phase; SP, mean percentile values of TGF-β expression in the endometrial tissue from four patients in the secretory phase; AT, mean percentile values of TGF-β expression in the endometrial tissue from five post-menopausal patients; EC, mean percentile values of TGF-β expression in the endometrial tissue from 13 patients affected by endometrial carcinoma; EP, mean percentile values of TGF-β expression in the tissue isolated from two patients affected by benign endometrial polyps.

expression of TGF-β1 mRNA in the different neoplastic (lanes 3–5) and non-neoplastic tissues (lanes 6, 8 and 9) in comparison with the RNA from samples of proliferative endometrium (lane 7) used as normal control. To normalize the differences due to mRNA loading and transfer, the same blots were dehybridized and rehybridized again with a human 28S rRNA cDNA probe. The values of TGF-β1 expression in all tissues were related to the 28S RNA expression accounting for the RNA loading in each sample and calculated as a percentage of control (normal proliferative tissue). Finally, the mean values of at least three separate measurements for each patient are reported in Figures 2 and 3.

*© Cancer Research Campaign 1998*

**Figure 4** TGF-β1 immunoreactivity in endometrial tissues. The mean values of TGF-β1 percentile immunoreactive cells are separately reported for the epithelial and stromal cells of the endometrial tissues from six patients in the proliferative phase (NP), four in the secretory phase of the menstrual cycle (SP), five post-menopausal patients (AT), 13 patients affected by endometrial carcinoma (EC) and two with endometrial polyps (EP). □, epithelium; ▨, stroma

As shown in Figure 2, a dramatic decrease of TGF-β1 steady-state level mRNA expression was detected in all cases of endometrial carcinoma compared with the samples of proliferative endometrium. The percentile reduction of TGF-β1 expression ranged from 9% up to 88%, with an average value of 42%. No direct correlations were found between the decrease in TGF-β1 expression and tumour differentiation; interestingly, the most reduced level of TGF-β1 mRNA expression was found in the poorly differentiated carcinoma of the serous type.

We also tested TGF-β1 expression in normal tissues from four patients in the secretive phase and from the five post-menopausal patients (Figure 3). We could detect a drastically decreased (32%) expression of TGF-β in secretory endometria compared with normal proliferative tissues. On the contrary, highly increased (300%) expression in the atrophic tissues was found.

Moreover, we also tested TGF-β1 mRNA expression in the two endometrial polyps obtained from a woman with proliferative endometrium and from a post-menopausal woman. A statistically significant ($P < 0.05$) increase (195%) of TGF-β1 mRNA expression was demonstrated in both samples.

### TGF-β1 – immunohistochemistry

The results of the semiquantitative evaluation of TGF-β1 immunoreactivity are schematically illustrated in Figure 4, and the corresponding mean values are reported in Table 1. No statistically significant correlations were found between the extent of TGF-β1 positivity in either the epithelial and stromal compartments (Figure 5) and the degree of tumour differentiation or the tumour histotype. Epithelial cells of endometrial carcinoma demonstrated the highest percentages of TGF-β1 immunoreactive cells in the whole series of cases, followed by the epithelial cells from endometria in the secretory, proliferative and post-menopausal phases. A trend for higher TGF-β1 positivity in both the stromal

**Table 1** TGF-β1 immunoreactivity in the endometrium

| Tissue | Glands $P < 0.005$ | Stroma $P < 0.25$ |
|---|---|---|
| NP ($n = 6$) | 0.50 (0.20) | 0.83 (0.15) |
| SP ($n = 4$) | 0.75 (0.41) | 1.00 (0.35) |
| AT ($n = 5$) | 0.40 (0.30) | 1.20 (0.20) |
| EC ($n = 13$) | 1.61 (0.28) | 1.08 (0.20) |
| EP ($n = 2$) | 1.00 (1.00) | 2.0 (0.71) |

The mean values of TGF-β1 percentile immunoreactive cells are reported in the glands and stromal compartment of the endometrial tissue. s.e.m. is reported in brackets. NP, proliferative endometria; SP, secretory endometria; AT, post-menopausal endometria; EC, endometrial carcinomas; EP, endometrial polyps.

**Figure 5** TGF-β1 immunoreactivity in stromal and neoplastic epithelial cells from endometrial adenocarcinoma. Alkaline phosphatase–anti-alkaline phosphatase (APAAP) anti-TGF-β1; 250×

and epithelial tumour cells was detected in the typical endometrioid histotype compared with mucus-producing, adenosquamous and serous carcinoma.

In tissue samples from women in their early/mid-proliferative phase, the percentage of TGF-β1-positive stromal cells (mean value = 0.83) was similar to that in the glandular epithelial cells (mean value = 0.5) (Figure 6).

Samples from women in the secretory phase demonstrated approximately the same percentage of TGF-β1-positive cells in stromal (mean value = 1) and epithelial endometrial cells (mean value = 0.75).

TGF-β1 immunoreactivity in tissue samples from post-menopausal women was detected in stromal endometrial cells more than in glandular epithelial cells: the mean value for TGF-β1-immunoreactivity in the stromal compartment of post-menopausal women was 1.25, whereas the mean value for the epithelial component was 0.5.

Figure 6 TGF-β1 immunoreactivity in normal endometrial glandular and stromal cells. Alkaline phosphatase–anti-alkaline phosphatase (APAAP) anti-TGF-β1; 250×

Figure 7 TGF-β1r immunoreactivity in endometrial tissues. The mean values of TGF-β1r percentile immunoreactive cells are separately reported for the epithelial and stromal cells of the endometrial tissues from six patients in the proliferative phase (NP), four in the secretory phase of the menstrual cycle (SP), five post-menopausal patients (AT), 13 patients affected by endometrial carcinoma (EC) and two with endometrial polyps (EP). □, epithelium; ▨, stroma

Overall, TGF-β1 immunoreactivity was comparatively higher in the stromal compartment than in the epithelial cells of post-menopausal, early/mid-proliferative and secretory endometria; only endometrial carcinoma epithelial tumour cells displayed higher percentages of TGF-β1 immunoreactivity than stromal cells. The highest percentages of TGF-β1-immunoreactive stromal cells were detected in the tissues from the two endometrial polyps, followed by the samples of post-menopausal women, endometrial carcinoma, secretory and proliferative endometria.

In all cases of the present series the immunopositivity was exclusively detected in the cell cytoplasm. Scattered inflammatory cells were present in atrophic and neoplastic endometria and occasionally demonstrated TGF-β1 immunoreactivity. These rare TGF-β1-positive inflammatory cells were not included in the semiquantitative evaluations.

### TGF-β1 receptor (TGF-β1r) – immunohistochemistry

The results of the semiquantitative evaluation of TGF-β1r immunoreactivity are schematically illustrated in Figure 7, and the corresponding mean values are reported in Table 2. As for TGF-β1, TGF-βlr immunoreactivity was exclusively detected in the cell cytoplasm.

The mean values for the percentages of TGF-βlr-positive cells were 0.77 and 1.61, respectively, in the stromal and in the epithelial compartment of endometrial carcinoma (Figure 8).

In early/mid-proliferative endometrial samples, TGF-βlr positivity mean value was 0.33 for the stromal component and 0 for the epithelial component.

No TGF-βlr positivity was found in the stromal compartment of secretory endometria (mean values = 0 in the stromal component and 0.25 in the epithelial component).

Overall, TGF-βlr immunoreactivity was predominantly detected in the stromal compartment compared with the epithelial component and in the groups of women in post-menopausal stage and in the early/mid-proliferative phase, whereas TGF-βlr-positivity of epithelial cells overcame the positivity in stromal cells in the groups of women with secretory endometrium and with endometrial carcinoma.

When comparing the mean values of the percentages of TGF-β1r-immunoreactive cells with those of TGF-β1-immunoreactive cells, the groups of women in post-menopausal stage and with endometrial carcinoma showed very similar mean values both in the epithelial and in the stromal compartments. In the groups of women in their early/mid-proliferative or secretory phases, the mean values of TGF-β1-immunoreactive cells were much higher than those of TGF-β1r-immunoreactive cells, both in the epithelial and in the stromal cells.

### DISCUSSION

In this paper we have shown decreased TGF-β1 mRNA expression in endometrial carcinoma compared with proliferative endometria. On the contrary, immunohistochemical data demonstrate increased protein expression in both stromal and epithelial cells in endometrial carcinoma.

Table 2 TGF-β1r immunoreactivity in the endometrium

| Tissue | Glands $P < 0.005$ | Stroma $P < 0.005$ |
|---|---|---|
| NP ($n = 6$) | 0.00 (0.00) | 0.33 (0.19) |
| SP ($n = 4$) | 0.25 (0.21) | 0.00 (0.00) |
| AT ($n = 5$) | 0.60 (0.20) | 1.00 (0.05) |
| EC ($n = 13$) | 1.61 (0.25) | 0.77 (0.12) |
| EP ($n = 2$) | 0.00 (0.00) | 1.00 (0.05) |

The mean values of TGF-β1r percentile immunoreactive cells are reported in the glands and stromal compartment of the endometrial tissue. s.e.m. is reported in brackets. NP, proliferative endometria; SP, secretory endometria; AT, post-menopausal endometria; EC, endometrial carcinomas; EP, endometrial polyps.

 © Cancer Research Campaign 1998

**Figure 8** TGF-β1r immunoreactivity in epithelial cells from endometrial adenocarcinoma. Alkaline phosphatase–anti-alkaline phosphatase (APAAP) anti-TGF-β1r; 250×

Although mRNA expression generally parallels the degree of translation, in our study the increase of TGF-β protein did not seem to be associated with RNA increase. This seems to suggest that alterations of TGF-β expression in carcinomas is a post-transcriptionally regulated event. As immunolocalization reveals sites of mature protein rather than the site of its synthesis, one may speculate that TGF-β is activated in the glands of endometrial carcinoma.

These results clearly indicate a down-regulated expression of TGF-β1, at least at the mRNA level in endometrial carcinoma.

The most widely studied role of TGF-β is its regulatory effect on cell proliferation in both normal and transformed tissues: recent studies have shown that although TGF-β1 is a potent inhibitor of epithelial cell proliferation in vitro, it is expressed at higher levels in several tumour types (McMahon et al, 1986; Truong et al, 1993; Christeli et al, 1996; Friess et al, 1994; Perlino et al, 1996). 'In situ' hybridization and immunohistochemical studies demonstrated that endometrial cells do express TGF-β mRNA and protein (Chegini et al, 1994); the expression of TGF-β mRNA was documented both in normal endometrial tissue (Marshburn et al, 1994) and in human carcinoma cell lines (Boyd and Kaufman, 1990). Nevertheless, the role of TGF-β in the aetiopathogenesis of endometrial proliferative conditions has not been clarified yet.

Our findings of down-regulated TGF-β1 mRNA in endometrial carcinoma is in agreement with the results of another study (Boyd and Kaufman, 1990), which demonstrated 'in vitro' an inverse correlation between TGF-β1 mRNA expression and tumour differentiation in different subtypes of an endometrial carcinoma cell line (HEC). In the study of Boyd and Kaufman (1990), TGF-β expression seemed to be inversely correlated with tumour differentiation as TGF-β mRNA was more expressed in the less differentiated subtype of endometrial carcinoma cells than in the most differentiated ones.

Although we were not able to detect significant 'in vivo' correlation between TGF-β1 mRNA expression and tumour grade, we did observe the lowest expression in the most aggressive (serous) and less differentiated (G3) endometrial carcinoma. This incongruence might be ascribed to the limited number of endometrial carcinomas included in the present series or to different mRNA expression in 'in vitro' and 'in vivo' conditions. In this context, it should be underlined that complex interactions among TGF-β1, steroid hormones and other cytokines may take place 'in vivo', thus justifying for these different results.

On the other hand, Gold et al (1994) have shown a statistically significant increase in 'in vivo' immunostaining for all three TGF-β isoforms in endometrial glandular epithelium in complex hyperplasia and carcinoma. The authors suggested a possible paracrine role of TGF-β in hyperplastic and malignant endometrial lesions. Our study lends further support to this hypothesis, although the results of these two studies are not in complete agreement. The discrepancies might be due, at least in part, to the different methods used in the two studies: Gold et al detected TGF-β1 mRNA expression by means of 'in situ' hybridization instead of Northern blotting analysis. Furthermore, that study quantitatively evaluated the intensity of TGF-β1 immunoreactivity, whereas we measured the percentage of TGF-β1-immunoreactive cells.

To verify the specificity of TGF-β1-altered expression we also measured mRNA levels in endometrial tissues obtained from patients with endometrial polyps. Interestingly, in such benign lesions the number of TGF-β1-positive cells was higher than in all other samples, and TGF-β1 mRNA expression was up-regulated in endometrial polyps compared with normal proliferative endometria. One could speculate that the down-regulated TGF-β1 expression is a specific effect of the endometrial tumoural condition.

Moreover, we investigated the role of this growth factor during the physiological changes of uterine tissues throughout the menstrual cycle. In agreement with other authors (Casey et al, 1996) we found a significantly increased TGF-β mRNA expression in post-menopausal women. Our immunohistochemical data showed a parallel increase of TGF-β1 and its receptor in the stromal compartment of atrophic tissues, thus indicating a key role of this growth factor in the control of cellular atrophy.

In this study we also found a statistically significant decrease of TGF-β1 mRNA expression in the secretory phase, whereas both TGF-β1 and TGF-β1r were strongly expressed especially in the epithelial component from the same patients. Other authors (Kauma et al, 1990 and Mashburn et al, 1994) have detected increased TGF-β expression in the late secretory and early proliferative phases. Our immunohistochemical data seem to confirm these results, thus indicating that during the late proliferative and the early/mid-secretory phase, a substantial increase in TGF-β protein expression may lead to growth arrest and induce the transition from cellular proliferation to differentiation of various endometrial cell types.

In conclusion, our study corroborates previous investigations that demonstrated a pivotal role of TGF-β1 in both physiological and neoplastic conditions of the endometrium. TGF-β1 seems to act as a negative regulator of cell growth in the transition from the proliferative to the secretory phase of the menstrual cycle and in endometrial atrophy. Moreover TGF-β seems to participate in the mechanisms of endometrial carcinogenesis, but its role and interactions with other regulatory molecules needs to be further elucidated.

© Cancer Research Campaign 1998

### ACKNOWLEDGEMENTS

The authors wish to thank Dr S Reshkin for helpful and stimulating discussion. This work was partially supported by CNR: PF 'FATMA'; by grants from the 'Associazione Italiana per la Ricerca sul Cancro' (AIRC) and from the 'Ministero per l'Università e la Ricerca Scientifica e Tecnologica' (60%).

### REFERENCES

Barnard JA, Lyons RM and Moses HI (1990) The cell biology of transforming growth factor β. *Biochim Biophys Acta* **1032**: 79–87

Berchuck A and Boyd J (1995) Molecular basis of endometrial cancer. *Cancer* (suppl.) **76**: 2034–2040

Bhatia P, Taylor WR, Greenberg AH and Wright JA (1994) Comparison of glyceraldehyde-3-phosphate dehydrogenase and 28S-ribosomal RNA gene expression as RNA loading controls for Northern blot analysis of cell lines of varying malignant potential. *Ann Biochem* **216**: 223–226

Boyd JA and Kaufman DG (1990) Expression of transforming growth factor β1 by human endometrial carcinoma cell lines: inverse correlation with effect on growth rate and morphology. *Cancer Res* **50**: 3394–3399

Casey ML and Macdonald PC (1996) The endothelin parathyroid hormone-related protein vasoactive peptide system in human endometrium: modulation by transforming growth factor β. *Human Reprod* **11**: 62–82

Chegini N, Zao Y, Williams RS and Flanders KC (1994) Human uterine tissue throughout the menstrual cycle expresses transforming growth factor β1 (TGF-β1), TGF-β2 and transforming growth factor-β3 and TGF-β Type II receptor for messenger ribonucleic acid and protein and contains [$^{125}$I]TGF-β1-binding sites. *Endocrinology* **135**: 439–449

Chirgwin JM, Przybyla AE, Raymond JM and Rutter WJ (1979) Isolation of biologically active ribonucleic acid from source enriched in ribonuclease. *Biochemistry* **18**: 5294–5299

Christeli E, Zoumpourlis V, Kiaris H, Ergazaki M, Vassilaros S and Spandidos DA (1996) TGF-β1 overexpression in breast cancer: correlation with clinicopathological data. *Oncol Reports* **3**: 1115–1118

Cline MJ (1996) Overview: the nature of malignancy. In *Apoptosis and Cell Cycle Control in Cancer*, Shaun N and Thomas B (eds), pp. 131–146. Latchman: London

Coffey RJ, Goustin AS, Soderquist AM, Shipley GD, Wolfshohl J, Carpenter G and Moses HL (1987) Transforming growth factor α and β expression in human colon cancer lines: implications for an autocrine model. *Cancer Res* **47**: 4590–4594

Derynck R, Jarret JA, Chen EY, Eaton DH, Bell JR, Assoian RK, Roberts AB, Sporn MB and Goeddel DV (1985) Human transforming growth factor-β complementary DNA sequence and expression in normal and transformed cells. *Nature* **316**: 701–705

Derynck R, Goeddel DV, Ullrich A, Gutterman JU, Williams RD, Bringman TS and Berger WH (1987) Synthesis of messenger RNAs for transforming growth factors α and β and the epidermal growth factor receptor by human tumors. *Cancer Res* **47**: 707–712

Feuerstein N and Cooper HL (1984) Studies of differentiation of promyelocytic cells by phorbol ester. *Biochim Biophys Acta* **781**: 239–246

Friess H, Yamanaka Y, Buchler M, Ebert M, Beger HG, Gold LI and Kore M (1994) Enhanced expression of transforming growth factor β isoforms in pancreatic cancer correlates with decreased survival. *Gastroenterology* **105**: 1846–1856

Giudice LC (1994) Growth factors and growth modulators in human uterine endometrium: their potential relevance to reproduction medicine. *Fertil Steril* **61**: 1–17

Gold LI, Saxena B, Mittal KR, Marmor M, Goswami S, Nactigal L, Korc M and Demoupoulos RI (1994) Increased expression of transforming growth factor β isoforms and basic fibroblast growth factor in complex hyperplasia and adenocarcinoma of the endometrium: evidence for paracrine and autocrine action. *Cancer Res* **54**: 2347–2358

Gurpide E (1991) Endometrial cancer: biochemical and clinical correlates. *J Natl Cancer Inst* **83**: 405–416

Jetten AM, Shirley JE and Stoner G (1986) Regulation of proliferation and differentiation of respiratory tract epithelial cells by TGF-β. *Exp Cell Res* **167**: 539–549

Kauma S, Matt D, Strom S, Eierma D and Turner T (1990) Interleukin-1b, human leukocyte antigen HLA-DRα and transforming growth factor-β expression in endometrium, placenta and placental membranes. *Am J Obstet Gynecol* **163**: 14130–14137

Kimchi A, Wang X-F, Weinberg RA, Cheietz S and Massagué J (1988) Absence of TGF-β receptor and growth inhibitory responses in retinoblastoma cells. *Science* **240**: 196–198

McMahon JB, Richards WL, del Campo AA, Song MK and Thorgiersson SS (1986) Differential effects of transforming growth factor-β on proliferation of normal and malignant rat liver epithelial cells in culture. *Cancer Res* **46**: 4665–4571

Marshburn PB, Arici AM and Casey ML (1994) Expression of transforming growth factor-β1 messenger ribonucleic acid and the modulation of deoxyribonucleic acid synthesis by transforming growth factor-β1 in human endometrial cells. *Am J Obstet Gynecol* **170**: 1152–1158

Massagué J (1990) The transforming growth factor-β family. *Annu Rev Cell Biol* **6**: 597–641

Murphy LJ, Gong Y and Murphy LC (1991) Growth factors in normal and malignant tissue. *Ann NY Acad Sci* **622**: 383–401

Niitsu Y, Urushizaki Y, Koshida Y, Terui K, Mahara K, Kohgo Y and Urushizaki I (1988) Expression of TGF-β gene in adult T-cell leukemia. *Blood* **71**: 263–266

Paulin C, Avallet O, Tardy F, Saez J, Fusco A and Fabien N (1995) Production of TGF-β and HGF receptor by a differentiated papillary thyroid carcinoma cell line (B-CPAP): implication in malignancy. *Int J Oncol* **7**: 657–660

Perlino E, Ciampolillo A, Maiorano E, Pannone E, Viale G, Giorgino R and Marra E (1996) Transforming growth factor-β1 (TGF-β1) expression in proliferating thyroid disease. *Int J Oncol* **9**: 83–88

Reiss M and Sartorelli AC (1987) Regulation of growth and differentiation of human keratinocyte by type β transforming growth factor and epidermal growth factor. *Cancer Res* **47**: 6705–6709

Roberts AB and Sporn MB (1990) The transforming growth factor-βs. In *Peptide Growth Factors and their Receptors*, Sporn MB and Roberts AB (eds), pp. 419–472. Springer: Heidelberg

Smith SK (1994) Growth factor in the human endometrium. *Human Reprod Update* **9**: 936–946

Tabibzadeh S (1991) Human endometrium: an active site of cytokine production and action. *Endocrinol Rev* **12**: 272–290

Tang X-M, Zhao Y, Rossi MY, Abu-Rustum RS, Ksander GA and Ghegini N (1994) Expression of Transforming Growth Factor-β (TGF-β) isoforms and TGF-β type II receptor messenger ribonucleic acid and protein, and the effect of TGF-βs on endometrial stromal cells growth and protein degradation in vitro. *Endocrinology* **135**: 450–459

Truong LD, Kadmon D, McCune BK, Flanders KC, Scardino PT and Thompson TC (1993) Association of transforming growth factor β1 with prostate cancer. *Human Pathol* **24**: 4–9

Van Noorden S (1986). Tissue preparation and immunostaining for light microscopy. In *Immunocytochemistry. Modern Methods and Applications*, Polak JM and Van Noorden S (eds), pp. 26–53. Wright: Bristol, UK

 © *Cancer Research Campaign 1998*